# Data in support of photosynthetic responses in a chromosome segment substitution line of ‘Khao Dawk Mali 105’ rice at seedling stage

**DOI:** 10.1016/j.dib.2018.09.128

**Published:** 2018-10-03

**Authors:** Panita Chutimanukul, Boonthida Kositsup, Kitiporn Plaimas, Teerapong Buaboocha, Meechai Siangliw, Theerayut Toojinda, Luca Comai, Supachitra Chadchawan

**Affiliations:** a Center of Excellence in Environment and Plant Physiology, Department of Botany, Faculty of Science, Chulalongkorn University, Bangkok 10300, Thailand; b AVIC Research Center, Department of Mathematics and Computer Science, Faculty of Science, Chulalongkorn University, Bangkok 10300, Thailand; c Department of Biochemistry, Faculty of Science, Chulalongkorn University, Bangkok 10300, Thailand; d Rice Gene Discovery Unit, National Center for Genetic Engineering and Biotechnology, Kasetsart University, Kamphaengsaen Campus, Nakhon Pathom 73140, Thailand; e Department of Plant Biology, UC Davis Genome Center, UC Davis, Davis, CA 95616, USA; f Omics Science and Bioinformatics Center, Faculty of Science, Chulalongkorn University, Bangkok 10300, Thailand

## Abstract

The rice chromosome segment substitution lines (CSSLs) of ‘Khao Dawk Mali 105’ (‘KDML105’) genetic background were developed by backcrossing with ‘KDML105’ rice and transferring the region from chromosome 1 of DH212 which was expected to contain the full putative salt tolerance genetic region. Line of CSSL11, CSSL12, and CSSL16 contained the full putative salt tolerance genetic region were evaluated with the parental lines, ‘KDML105’ and DH212 at seedling stage of rice. The physiological responses in rice plants were grown under normal condition and 75 mM of NaCl, and then comparative photosynthetic parameters, chlorophyll fluorescence parameters, PhiPS2, ETR, NPQ, as well as growth analysis. In this article, the data of physiological response evaluation in rice at seedling stage after salt stress treatment can be found. This can be useful as the information of the photosynthesis response to salt stress to other rice cultivars and related species.

**Specifications table**

**Table:** 

Subject area	*Biology*
More specific subject area	*Plant science*
Type of data	*Figure and tables*
How data was acquired	*Photosynthesis parameters and light reaction process activities were acquired by using LI-6400 XT portable photosynthesis system (LI-COR, Lincoln, NE, USA) and chlorophyll fluorescence parameters were obtained by using FMS 2 fluorescence monitoring system (Hansatech, King׳s Lynn, UK). Balance was used to weigh the plant materials.*
Data format	*Analyzed data*
Experimental factors	*Seeds of three CSSL lines and their parents (KDML105 and DH212) were germinated and soil grown under natural condition for 21 days, then, they were separated into two groups one was grown in normal condition, while the other was treated with 75 mM NaCl solution for 9 days.*
Experimental features	*Complete randomized design (CRD) with 4 replications was performed to compare the difference in each parameter collected. Each replicate consisted of 3 plants. The measurement included photosynthetic parameters (photosynthetic rate, stomatal conductance, internal CO*_*2*_*concentration, and transpiration rate), chlorophyll fluorescence parameters (F_v_/F_m_, PhiPS2, ETR, and NPQ), fresh weight and dry weight when plants were grown in normal and salt stress condition.*
Data source location	*Nakhon Pathom, Thailand*
Data accessibility	*Data is provided this article and also in the supplementary with the article entitled photosynthetic responses and identification of salt tolerance genes in a chromosome segment substitution line of ‘Khao Dawk Mali 105’ rice.*
Related research article	*Chutimanukul et al., 2018. Photosynthetic responses and identification of salt tolerance genes in a chromosome segment substitution line of ‘Khao Dawk Mali 105’ rice. Environ. Exp. Bot. in press.*

**Value of the data**

• The data are valuable for understanding the response of photosynthesis process of rice at seedling stage under salt stress.
• The data provide the detail information of growth analysis fresh and dry weight at seedling stage.
• The data of the chlorophyll fluorescence parameters, Fv/Fm, PhiPS2, ETR, and NPQ of rice under salt stress show the adaptation of the photosynthetic system in the tolerance lines of rice at seedling stage. This can be benchmark for the similar studies in other rice cultivars or related species.

## 1 Data

Data describe performance of rice seedlings of 3 chromosome segment substitution lines (CSSL), CSSL11, CSSL12, CSSL16 and parental lines, ‘KDML105’ and DH212 rice under normal and salt stressed conditions. The performance includes fresh weight and dry weight of above ground tissues, maximum photosynthesis rate (*A*_*max*_), stomatal conductance (*g*_*s*_), intercellular CO_2_ concentration (Ci), transpiration rate (Trans), *F_v_/F_m_*, PhiPS2, ETR, and NPQ.

## 2 Experimental design, materials, and methods

### 2.1 Experimental design

The chromosome segment substitution lines (CSSLs) of ‘Khao Dawk Mali 105’ (‘KDML105’) were developed, by transferring the region between the RM1003 and RM3362 markers on chromosome 1 from DH212 which was expected to contain the putative drought tolerance genetic region [1], [2]. Three CSSL lines, CSSL11, CSSL12 and CSSL16 were compared with ‘KDML105’ and DH212 under normal and salt stress conditions at seedling stage. The physiological responses of treated plants were evaluated at four time points in each condition and were conducted using four replicates, each consisting of 3 plants, and a completely randomized design. For growth analysis, the above-ground shoot fresh and dry weights were determined at the end of the experiment. Analysis of variance (ANOVA) was used to evaluate the data for each parameter. The means were compared with Duncan׳s multiple range tests (DMRT) using the IBM SPSS Statistics program (*P* < 0.05 was used as the threshold for significance).

## 3 Materials and methods

### 3.1 Plant materials

CSSL11, CSSL12, and CSSL16 seeds with a ‘KDML105’ genetic background and their DH212 and ‘KDML105’ as their parents were provided from the Rice Gene Discovery Unit, National Center for Genetic Engineering and Biotechnology. CSSL11, CSSL12, and CSSL16 included the full segment containing the putative drought tolerance genes between RM1003 and RM3362 on chromosome 1 of DH212 [2].

### 3.2 Growth conditions

All rice seeds were germinated until roots were visible in plastic cups that contain distilled water for 5 days, the which they were transplanted into clay soil in pots with diameter of 25 cm and 40 cm of pot high. Clay soil was filled to ¾ capacity in the supplied pot. Pots were placed in the cement tank that can be flooded with nutrient solution or nutrient solution supplemented with NaCl solution for salt stress treatment. During flooding with Bangsai nutrient solution (1:100), rice plants were constantly supplied with fresh air by an aquarium pump. The nutrient solution consists of 50 g/L MgSO4, 80 g/L KNO_3_, 12.5 g/L NH_4_H_2_PO_4_, 8.5 g/L KH_2_PO_4_, 0.4 g/L Mn-EDTA, micronutrient 0.8 g/L, 100 g/L Ca(NO_3_)_2_, and 3 g/L Fe-EDTA. Fourteen-day old plants were treated with nutrient solution supplemented with 75 mM NaCl to simulate salt stress conditions (i.e., soil salinity stress with an electrical conductivity (EC) value of 7.8–8.2 dS m^−1^), EC was directly determined at 5 points in each pond and EC value was verified and adjusted, if needed, every day. The control plants were grown normally and treated with a nutrient solution lacking NaCl. Soil EC value for the controls was 1.1–1.2 dS m^−1^. The complete block design (CRD) with 4 replications (three plants per replication) was used to evaluate the different responses to salt stress. Plant seedlings were treated for 9 days. Fresh weight and dry weight of the above ground tissues were shown in Tables 1 and 2.

**Table 1:** Mean value of shoot fresh weight under normal condition for 9 days.

**Weight**	**Rice cultivars**					***F*-Test**
	**CSSL11**	**CSSL12**	**CSSL16**	**KDML105**	**DH212**	
**Fresh weight (g)**	2.461 ± 0.087^a^	1.848 ± 0.073^b^	2.063 ± 0.042^a^	2.122 ± 0.035^a^	1.998 ± 0.076^ab^	*
**Dry weight (g)**	0.351 ± 0.015^ab^	0.314 ± 0.015^b^	0.335 ± 0.008^b^	0.381 ± 0.012^a^	0.34 ± 0.008^b^	*

Data are shown as the mean ± SE, derived from 4 repeats. Means in a column with a different superscript lowercase letter are significantly different (*p* < 0.05; DMRT).

* Represent significant differences in CSSL lines KDKL105 and DH212.

**Table 2:** Mean value of shoot fresh weight under 75 mM NaCl for 9 days.

**Weight**	**Rice cultivars**					***F*-Test**
	**CSSL11**	**CSSL12**	**CSSL16**	**KDML105**	**DH212**	
**Fresh weight (g)**	1.383 ± 0.052^a^	1.137 ± 0.036^b^	1.308 ± 0.023^a^	1.366 ± 0.011^a^	1.222 ± 0.094^ab^	*
**Dry weight (g)**	0.278 ± 0.003^a^	0.197 ± 0.007^b^	0.262 ± 0.004^a^	0.24 ± 0.006^ab^	0.261 ± 0.008^a^	*

Data are shown as the mean ± SE, derived from 4 repeats. Means in a column with a different superscript lowercase letter are significantly different (*p* < 0.05; DMRT).

* Represent significant differences in CSSL lines KDKL105 and DH212.

### 3.3 Physiological parameters measurement

**Fig. 1:**
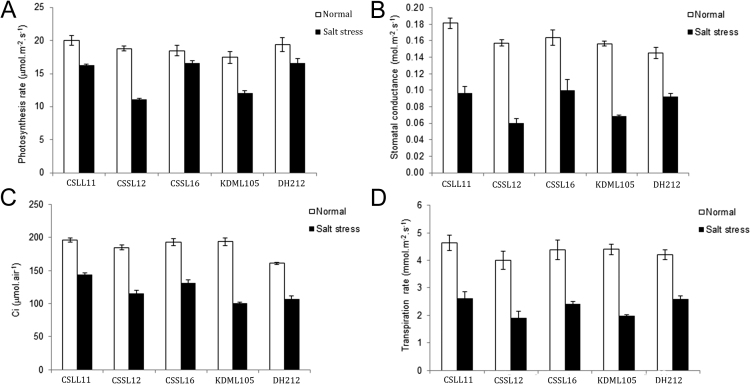
Photosynthesis rate (A), stomatal conductance (B), internal CO_2_ concentration (C) and transpiration rate (D), of CSSL11, CSSL12, CSSL16, ‘KDML105’, and DH212 plants at the seedling stage when grown in normal and salt stress conditions.

**Fig. 2:**
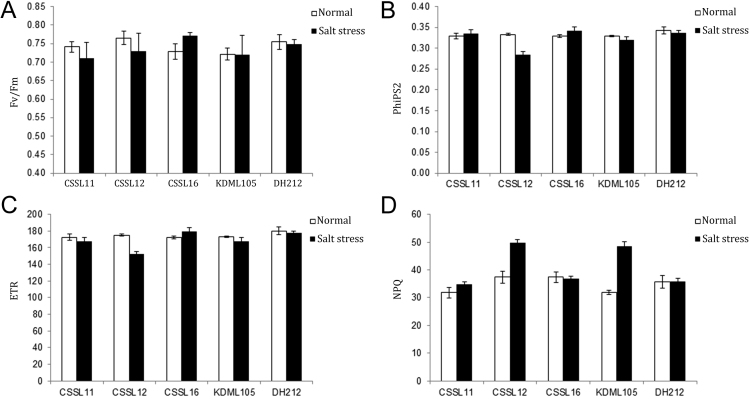
Fv/Fm (A), PhiPS2 (B), ETR (C) and NPQ (D), of CSSL11, CSSL12, CSSL16, ‘KDML105’, and DH212 plants at the seedling stage when grown in normal and salt stress conditions.

To evaluate the physiological responses at seedling stage: photosynthetic parameters and chlorophyll fluorescence parameters of treated plants, the following characteristics were evaluated on days 0, 3, 6, and 9 after treatment. Gas exchange was measured during the same period with a LI-6400 XT portable were evaluated including *A*_*max*_*, g*_*s*_*,* Ci, Trans (Fig. 1). Chlorophyll fluorescence parameters including *F_v_*/*F_m_* was measured using the FMS 2 fluorescence monitoring system (Hansatech, King׳s Lynn, UK) according to the recommended procedures while PhiPS2, and ETR were determined by LI-6400 XT. NPQ was calculated from (*F_m_*/*F_m′_*)−1 [3] (Fig. 2). Rice leaves were dark-adapted for 40 min with leaf clips. The middle portions of the uppermost fully expanded leaves were analyzed using the following conditions: molar flow of air per unit leaf area: 500 mmol l^−1^ m^−2^ s^−1^; leaf surface photosynthetically active radiation: 1200 mol m^−2^ s^−1^; leaf temperature: 30.0–37.0 °C; and CO_2_ concentration: 380.0 mol mol^−1^. For photosynthesis parameters, the measurement was performed on the youngest fully expanded leaves, and each measurement was done on 3 positions of the leaf and the average was used for the analysis. Fv/Fm, PhiPS2, ETR, and NPQ were analyzed. The data with statistical analysis of the normal grown seedlings were shown in Supplementary data 1 and the data from salt stressed seedlings are in Supplementary data 2.

## References

[1] Toojinda T., Siangliw J.L., Punyawaew K., Kanjoo V. (2011). Development of Single QTL Near Isogenic Lines (NILs) of KDML105 for Drought Tolerance. Pathum Thani, Research Report.

[2] Chutimanukul P., Kositsup B., Plaimas K., Buaboocha T., Siangliw M., Toojinda T., Chadchawan S. (2018). Photosynthetic responses and identification of salt tolerance genes in a chromosome segment substitution line of ‘Khao dawk mali 105’ rice. Environ. Exp. Bot..

[3] Bilger W., Bjorkman O. (1990). Role of the xanthophylls cycle in photoprotection elucidated by measurements of light-induced absorbance changes, fluorescence and photosynthesis in Hedera canariensis. Photosynth. Res..

