# Supplementary material for: Data in support of photosynthetic responses in a chromosome segment substitution line of ‘Khao Dawk Mali 105’ rice at seedling stage
Source: Data Brief. 2018 Oct 3;21:307–12. doi: 10.1016/j.dib.2018.09.128 (PMC6197707; doi:10.1016/j.dib.2018.09.128)
Supplement: Supplementary file 1 — Supplementary material [file mmc1.docx]

**Declaration of interest**

**All authors have no conflicts of interest to publish this data. A**ll authors have approved the final version of the manuscript being submitted.
